# Complete Genome Sequence of Acinetobacter baumannii Strain KBN10P04593, a Clinical Isolate from South Korea

**DOI:** 10.1128/mra.01032-22

**Published:** 2023-02-22

**Authors:** Md. Maidul Islam, Jeongah Kim, Kyeongmin Kim, Je Chul Lee, Minsang Shin

**Affiliations:** a Department of Microbiology, School of Medicine, Kyungpook National University, Jung-gu, Daegu, South Korea; Loyola University Chicago

## Abstract

Acinetobacter baumannii is an opportunistic nosocomial pathogen that is responsible for various life-threating infections in immunocompromised hosts. We present the complete 3.93-Mb genome sequence of A. baumannii KBN10P04593, generated by combining PacBio and Illumina technologies.

## ANNOUNCEMENT

Acinetobacter baumannii is a Gram-negative pathogen that is also known as a member of the ESKAPE (Enterococcus faecium, Staphylococcus aureus, Klebsiella pneumoniae, Acinetobacter baumannii, Pseudomonas aeruginosa, and Enterobacter species) pathogens, which are important multidrug-resistant (MDR) microorganisms in health care systems worldwide (1, 2). The capability of A. baumannii to acquire and spread multidrug and even pandrug resistance phenotypes is a major threat to public health (3, 4). Understanding the mechanisms of antimicrobial resistance and developing new antimicrobials for this MDR pathogen are urgently required. In this study, we report the genome sequence of the clinical strain A. baumannii KBN10P04593.

The clinical isolate A. baumannii KBN10P04593 was obtained from the Kyungpook National University Chilgok Hospital Culture Collection for Pathogens (Daegu, South Korea). The isolate was recovered from a blood sample from a 63-year-old patient admitted to Kyungpook National University Chilgok Hospital (Daegu City). The isolate was confirmed by microbiological standard culture and biochemical tests and PCR assay. This study did not require institutional review board (IRB) approval since it was performed on bacterial isolates that did not include patient protected health information. Genomic DNA was isolated from overnight Luria-Bertani broth cultures from a single colony grown at 37°C with shaking, using the Exgene cell SV kit (GeneAll, Seoul, Republic of Korea) according to the manufacturer’s protocol. The DNA quantity and quality were assessed using a NanoDrop 2000 spectrometer (Thermo Scientific, Wilmington, USA) and running the sample on a 1% agarose gel for electrophoresis.

The complete genome of KBN10P04593 was sequenced using the Pacific Biosciences (Menlo Park, CA) RS II platform and Illumina (San Diego, CA) MiSeq platform at Macrogen Inc. (Seoul, South Korea). For PacBio sequencing, the SMRTbell template was used, following the standard PacBio 20-kb library preparation procedure (5). For Illumina, a sequencing library was prepared by random fragmentation of the DNA sample, followed by 5′ and 3′ adapter ligation, and the sequencing data were used for curation of the raw data for further analysis. *De novo* assembly of the raw reads was performed using Canu v1.7 (https://canu.readthedocs.io/en/stable/index.html) (6), and after assembly, the Illumina reads were used to correct errors by correcting bases, fixing misassemblies, and filling gaps using Pilon v1.21 (https://github.com/broadinstitute/pilon/wiki) (7). The NCBI Prokaryotic Genome Annotation Pipeline (PGAP) v6.1 was used for annotation (8).

From the raw data, a total of 81,170 subreads (*N*_50_, 11,210 bp) were generated using the PacBio platform; from the Illumina paired-end sequencing, a total of 6,811,588 reads (2,665,998 Illumina reads) were used for processing. The assembly consisted of a single circular chromosome, with a length of 3,931,555 bp and an average G+C content of 39.1%, with 102.0× genome coverage. In addition, the genome encodes 3,794 predicted genes, including 3,635 protein-coding sequences (CDSs), 18 rRNAs, 73 tRNAs, 5 noncoding RNAs (ncRNAs), and 63 pseudogenes. From the sequencing results, the following antimicrobial resistance determinants were identified using CARD (9) (https://card.mcmaster.ca/): efflux pumps (*adeAC*, *adeFGH*, and *adeIJK*) and their regulators (*adeL*, *adeN*, and *adeR*), *bla*_OXA-23_, *bla*_OXA-66_, AbaF, ADC-30, *abeS*, AbaQ, LpsB, AmvA, *parC*, *ant(3″)-IIc*, and *gyrA*. Several genes belonging to eight different classes of virulence factors (adherence, biofilm formation, enzymes, immune evasion, iron uptake, quorum sensing, serum resistance, and stress adaptation) were detected in the genome after searching the Virulence Factor Database (VFDB) (10).

The purpose of publishing the complete genome sequence of the clinical strain KBN10P04593 is to carry out further studies regarding MDR A. baumannii strains found in hospitals throughout the world.

### Data availability.

The complete genome sequence of A. baumannii strain KBN10P04593 has been deposited at GenBank under accession number CP099989, BioProject accession number PRJNA853386, and BioSample accession number SAMN29386244. The version described in this paper is the first version, CP099989.1. The PacBio and Illumina raw data are accessible at the SRA under the accession numbers SRX18010182 (PacBio) and SRX17677858 (Illumina).
